# Dual Targeting of Orphan Nuclear Receptors NR4A1 and NR4A2 for Nonhormonal Endometriosis Therapy

**DOI:** 10.1210/endocr/bqaf144

**Published:** 2025-09-26

**Authors:** Wai Ning Tiffany Tsui, Yuri Park, Srijana Upadhyay, Da Mi Kim, Lei Zhang, Gus Wright, Amanuel Hailemariam, Arafat Rahman Oany, Sang Jun Han, Stephen Safe

**Affiliations:** Department of Veterinary Physiology and Pharmacology, College of Veterinary Medicine, Texas A&M University, College Station, TX 77843, USA; Department of Molecular & Cell Biology, Baylor College of Medicine, Houston, TX 77030, USA; Department of Veterinary Physiology and Pharmacology, College of Veterinary Medicine, Texas A&M University, College Station, TX 77843, USA; Department of Veterinary Pathobiology, College of Veterinary Medicine, Texas A&M University, College Station, TX 77845, USA; Department of Veterinary Physiology and Pharmacology, College of Veterinary Medicine, Texas A&M University, College Station, TX 77843, USA; Department of Veterinary Pathobiology, College of Veterinary Medicine, Texas A&M University, College Station, TX 77845, USA; Department of Veterinary Physiology and Pharmacology, College of Veterinary Medicine, Texas A&M University, College Station, TX 77843, USA; Department of Veterinary Physiology and Pharmacology, College of Veterinary Medicine, Texas A&M University, College Station, TX 77843, USA; Department of Molecular & Cell Biology, Baylor College of Medicine, Houston, TX 77030, USA; Department of Veterinary Physiology and Pharmacology, College of Veterinary Medicine, Texas A&M University, College Station, TX 77843, USA

**Keywords:** NR4A1, NR4A2, DIM-3, 5-Br_2_, DIM-3, 5-Cl_2_, DIM-3-Cl-5-CF_3_, endometriosis

## Abstract

Previous studies show that orphan nuclear receptor 4A1 (NR4A1) regulates endometriotic cell growth, survival, estrogen receptor β (ERβ), mechanistic target of rapamycin signaling and fibrosis. NR4A2 is also expressed in epithelial and stromal derived endometriotic cells, and in this study the effects of 1,1-bis(3′-indolyl)-(3,5-disubstitutedphenyl)methane (DIM-3,5) dual NR4A1/nuclear receptor 4A2 (NR4A2) ligands and knockdown of NR4A1 and NR4A2 were investigated. The dual NR4A1/2 DIM-3,5 analogs inhibited previously identified proendometriotic pathways and gene products, and they also inhibited TWIST1 and multiple markers associated with epithelial-to-mesenchymal transition (EMT). The results show that both NR4A1 and NR4A2 regulate the same pathways, including endometriotic cell growth, survival, and migration and also some of the same genes in endometriotic epithelial and stromal cells. For example, DIM-3,5 compounds downregulate ERβ in stromal but not epithelial endometriotic cells, and this response is NR4A1- and not NR4A2-dependent. Among the EMT-related markers, claudin-1 is induced by DIM-3,5 ligands and after knockdown of NR4A1 or NR4A2 in both epithelial and stromal cells. Most of the EMT markers are downregulated by DIM-3,5 ligands and are coregulated by NR4A1 and NR4A2. In vivo studies showed that DIM-3,5-Cl_2_ significantly reduced the growth of endometriotic lesions in a mouse model without inducing cytotoxicity during treatment. Thus, DIM-3,5 derivatives simultaneously suppress NR4A1- and NR4A2-dependent endometriosis progression effectively and represent a promising nonhormonal therapeutic strategy to replace current hormone-based treatments that can be associated with adverse effects.

Endometriosis is an estrogen-dependent inflammatory disease that affects reproductive-aged women, and approximately 5.5 million women in the United States and 176 million worldwide suffer from symptoms such as pelvic pain and infertility (1-4). Endometriosis develops through the outgrowth of endometrial cells outside the uterine cavity, affecting areas such as the pelvic cavity, peritoneal surfaces, ovaries, ligaments, bowel, and bladder. Although several hypotheses have been proposed, the molecular etiology of endometriosis remains poorly defined (5). Antiestrogen and anti-inflammatory drugs have been used to treat endometriosis symptoms, based on the role of estrogen signaling and inflammatory responses for its progression (6, 7). For example, progestins, oral contraceptives, and GnRH antagonists have been used as hormonal therapies for the treatment of endometriosis to systemically suppress estrogen signaling (8-14). While hormonal therapy offers benefits, it is associated with severe adverse effects in endometriosis patients, including bone loss, blood clots, severe hot flushes, menstrual cycle and ovulation disruption, and hypertension (15, 16). Therefore, hormonal therapies are not viable long-term options for women who hope to conceive (3). There is a growing demand to develop new therapeutic strategies for endometriosis that offer high treatment efficacy with minimal adverse effects.

One of the major challenges in identifying druggable targets for endometriosis treatment is the lack of mechanistic information underlying the disease, largely due to its complexity. Numerous studies have been conducted to address this question. In addition to estrogen signaling, activation of inflammatory responses by differentially infiltrating immune cells, along with increased proliferation, survival signaling, angiogenesis, cell adhesion, and suppression of apoptosis, have an essential role in endometriosis progression (3, 4, 17-20). Therefore, targeting endometriosis-driving pathways has been considered and employed to overcome the severe adverse effects associated with hormonal therapy. For example, nonsteroidal anti-inflammatory drugs have been investigated for their ability to decrease inflammation and pain by inhibiting cyclooxygenase enzymes (21). N-palmitoylethanolamine has also been used to reduce endometriosis-associated inflammation by modulating mast cell activity (22). TGFβ plays a critical role in the progression of endometriosis (23) as its overexpression is strongly correlated with human endometriotic lesions (24-27). Moreover, in TGFβ1-null mice, the growth of endometriotic lesions is significantly decreased (28). Resveratrol is used to inhibit the TGFβ/ERK signaling pathways in endometriotic lesions, thereby reducing TGFβ-mediated fibrosis while also suppressing angiogenesis and exerting antioxidant effects (29). Etanercept, a TNF-α antagonist, is used to inhibit TNF-α–mediated inflammation in endometriotic lesions (30). Although several nonhormonal drugs have shown suppressive effects on endometriosis progression and symptom relief in preclinical models, no nonhormonal drug has been specifically approved for the treatment of endometriosis based on current evidence.

In addition to estrogen receptors, other nuclear receptors have been implicated in the progression of endometriosis. Our previous study revealed elevated levels of the orphan nuclear receptor NR4A1 in endometriotic lesions compared to normal endometrium, and NR4A1 was shown to promote endometriosis-driving cellular pathways, including cell growth, migration, survival, and fibrosis, in human endometrial cells (31, 32). Therefore, targeting NR4A1 is considered a nonhormonal therapeutic strategy for treating endometriosis. NR4A1 ligands including bis-indole derived compounds and flavonoids such as quercetin and kaempferol act as inverse agonists and inhibit endometriosis, driving NR4A1-dependent pathways (31, 32). In addition to NR4A1, NR4A2 might have an essential role in endometriosis progression along with NR4A1 due to high levels of NR4A2 in mesenchymal stem cells from menstrual flow of endometriosis patients (33) and in granulosa cells from patients with endometriosis (34). In cancer progression, both NR4A1 and NR4A2 exhibit pro-oncogenic activities (35). However, the role of NR4A2 and its interactions with NR4A1 in endometriosis progression has not been investigated. In this study, we have analyzed and compared the roles of NR4A1 and NR4A2 in endometriosis progression using RNA interference assays in human immortalized endometrial cells. Furthermore, we have developed novel compounds that bind to both NR4A1 and NR4A2, acting as inverse agonists to suppress their activity (36-39). These dual NR4A1/NR4A2 ligands effectively inhibited endometriosis progression in both cell culture and a mouse model.

## Materials and Methods

### Mouse and Human Subjects

All human sample collections were performed in accordance with the ethical standards of the institutional review board at Baylor College of Medicine, with written informed consent obtained from all participants prior to inclusion in the study (IRB Protocol #: H-50114). All animal studies were reviewed and approved by the Institutional Animal Care and Use Committee (IACUC) of Baylor College of Medicine and conducted in compliance with institutional and national guidelines for the care and use of laboratory animals (IACUC Protocol #: AN-5284, Assurance number: D16-00475). Only female mice were used in this study.

### Human Endometriotic Cells from Endometriosis Patients

Patient-derived immortalized human endometriotic epithelial cells (IHEEC) and human endometriotic stromal cells (IHESC), obtained from the laboratory of Dr. Sang Jun Han, were used in this study. Cells were cultured in Dulbecco's Modified Eagle Medium/Nutrient Mixture F-12 (DMEM/F12) supplemented with 10% fetal bovine serum (FBS). Cultures were maintained at 37 °C in a humidified incubator with 5% CO_2_ and 95% air.

### Antibodies

The primary antibodies used for Western blotting were as follows: cleaved poly(ADP-ribose) polymerase (C-PARP) (#5625S, RRID:AB_10699459), β1-integrin (#9699S, RRID:AB_11178800), claudin-1 (#13255T, RRID:AB_2798163), Vimentin (#5741T, RRID:AB_10695459), Slug (#9585T, RRID:AB_2239535), β-catenin (#8480T, RRID:AB_11127855), N-cadherin (#13116T, RRID:AB_2687616), ZO-1 (#8193T, RRID:AB_10898025), ZEB1 (#3396T, RRID:AB_1904164), Snail (#3879T, RRID:AB_2255011), phospho-mechanistic target of rapamycin (mTOR) (#2971S, RRID:AB_330970), mTOR (#2972S, RRID:AB_330978), and epidermal growth factor receptor (EGFR) (#4267T, RRID:AB_2895042), all from Cell Signaling Technology (Danvers, MA, USA); NR4A2 (#sc-376984X, RRID:AB_2893391), TWIST (#sc-81417, RRID:AB_1130910), and estrogen receptor β (ERβ, #sc-8974, RRID:AB_2102246) from Santa Cruz Biotechnology (Dallas, TX, USA); NR4A1 (#ab283264, RRID:AB_3665433) from Abcam (Waltham, MA, USA); β-actin (#A1978, RRID:AB_476692) from Sigma-Aldrich (Milwaukee, WI, USA); and α-smooth muscle actin (α-SMA, #GTX100034, RRID:AB_1240408), collagen type I α1 (COL1A1, #GTX112731, RRID:AB_10721155), connective tissue growth factor (CTGF, #GTX124232, RRID:AB_11169640), and fibronectin (FN, #GTX112794, RRID:AB_1950298) from GeneTex (Irvine, CA, USA); caspase 3 (Cleaved Asp175, #PA5-114687, RRID:AB_2899323) from Thermo Fisher Scientific (Waltham, MA, USA) was used for IHEEC cell line while cleaved caspase 3 (#25128-1-AP, RRID:AB_3073913) from Proteintech (Rosemont, IL, USA) was used for IHESC cell line. Secondary antibodies for rabbit (#7074, RRID:AB_2099233) and mouse (#7076, RRID:AB_330924) were purchased from Cell Signaling Technology.

For immunofluorescence staining, the following primary antibodies were used: TWIST1 (#90445T, RRID:AB_3064916) from Cell Signaling Technology and COL1A1 (#GTX112731, RRID:AB_10721155) and N-cadherin (#GTX127345, RRID:AB_2885644) from GeneTex.

For immunohistochemistry staining, antibodies against NR4A1 (#NB100-56745, RRID:AB_2153757) and NR4A2 (#AF2156, RRID:AB_2153894) from Novus Biologicals (Centennial, CO, USA) and Ki-67 (#ab16667, RRID:AB_302459) from Abcam were used.

### RNA Interference

IHEEC and IHESC cells were seeded in 6-well plates at a density of 1.0 × 10⁵ cells/well and cultured for 24 hours until reaching approximately 60% to 70% confluency. Cells were then transfected with 100 nM small interfering RNAs (siRNAs) per well using 7.0 μL of Lipofectamine™ RNAiMAX transfection reagent (#13778150, Thermo Fisher Scientific), according to the manufacturer's protocol. After 72 hours, cells were harvested for analysis of protein expression and apoptosis. siRNAs targeting NR4A1 (siNR4A1) and NR4A2 (siNR4A2) were purchased from Sigma-Aldrich and GenScript (Piscataway, NJ, USA), respectively. A nontargeting negative control siRNA (IgL2) was obtained from Qiagen (Montreal, ON, Canada). The siRNA sequences used in this study were as follows:

siNR4A1_1: Sigma, SASI_Hs02_00333289
siNR4A1_2: Sigma, SASI_Hs02_00333290
siNR4A2_1: GenScript, SC1518-RNAi_NR4A2_si1
siNR4A2_2: GenScript, SC1518-RNAi_NR4A2_si2

### Cell Proliferation and Migration Assays

Cell proliferation was assessed using the XTT Cell Viability Kit (Cell Signaling Technology) following the manufacturer's instructions. IHEEC and IHESC cells were seeded in 96-well plates at a density of 1.0 × 10⁴ cells/well in DMEM/F12 medium supplemented with 10% FBS and allowed to attach for 24 hours. The culture medium was then replaced with DMEM/F12 containing 2.5% charcoal-stripped FBS and treated with either vehicle control [dimethyl sulfoxide (DMSO)] or selected concentrations of DIM-3,5 analogs dissolved in DMSO. After 24 and 48 hours of treatment, 35 μL of the XTT reaction mixture comprising sodium 3′-[1-(phenylaminocarbonyl)-3,4-tetrazolium]-bis(4-methoxy-6-nitro)benzenesulfonic acid hydrate and N-methyl dibenzopyrazine methyl sulfate in a 50:1 ratio was added to each well. Plates were incubated for 4 hours at 37 °C, and absorbance was measured at 450 nm using a microplate reader. Experiments were performed and repeated in at least 3 independent experiments. Percent proliferation and IC_50_ values were calculated using GraphPad Prism version 10 (GraphPad Software, San Diego, CA, USA). IHEEC and IHESC cells (2 × 10^5^) were seeded in 6-well plates and allowed to adhere for 24 hours to reach approximately 80% confluency. A scratch was introduced using a sterile pipette tip, followed by treatment with selected DIM-3,5 analogs at 6.5 µM or transfection with siRNAs. Cell migration into the wound area was assessed 24 hours posttreatment. Images of the wound gap were captured before and after treatment using an AMG EVOS fluorescence microscope. The wound area was quantified using the Wound Healing Size Tool in ImageJ/Fiji (RRID:SCR_002285).

### Western Blotting

IHEEC and IHESC cells (2 × 10⁵) were seeded onto 6-well plates and allowed to adhere for 24 hours. Cells were then treated with DMSO (vehicle control) or various concentrations of selected DIM-3,5 analogs for 24 hours or transfected with siRNAs for 72 hours. Whole-cell lysates were prepared using RIPA buffer (#89901, Thermo Fisher Scientific) supplemented with protease and phosphatase inhibitors (#P3100 and #P3200, GenDEPOT, Barker, TX, USA). Protein concentrations were determined using the Bradford assay, and 25 µg of total protein per sample were resolved by SDS-PAGE and transferred onto polyvinylidene fluoride membranes by wet transfer. After blocking, membranes were incubated with primary and secondary antibodies, followed by washes. Detection was performed by treating the membranes with Immobilon Western Chemiluminescence HRP Substrate (#WBKLS0500, Millipore Sigma, Burlington, MA, USA), and the chemiluminescent signals were visualized using the Bio-Rad ChemiDoc MP Imaging System (Bio-Rad, Hercules, CA, USA). For both the siRNA knockdown and DIM-3,5 treatment experiments, the same lysates were used across multiple Western blots targeting different pathways. Consequently, β-actin loading controls may appear identical or highly similar across different figure panels. One-way ANOVA followed by Dunnett's post hoc test was used for statistical analysis of all Western blot quantification data obtained using ImageJ/Fiji, including comparisons involving 2 or more groups, to ensure consistency across datasets.

### Annexin V Staining and Flow Cytometry

**Figure 1.**
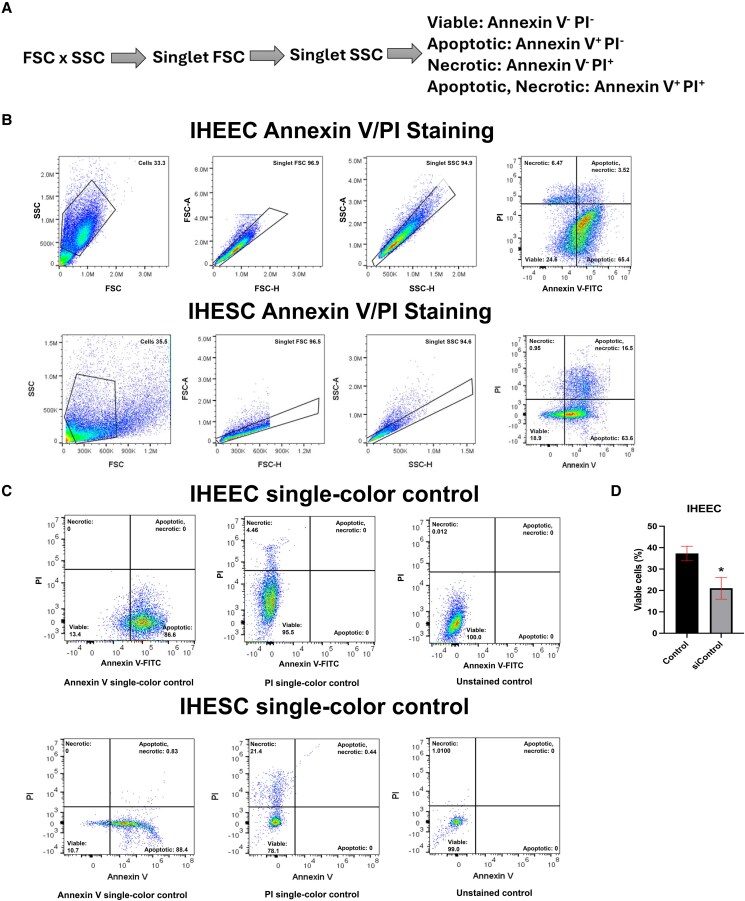
Annexin V staining flow gating strategy and comparison of lipofectamine RNAiMAX effects on cell viability on IHEEC. (A) Flow cytometry gating strategy for Annexin V/PI staining in IHEEC and IHESC cells. (B) Representative flow cytometry scatter plots for apoptosis analysis in both cell lines. (C) Single-color controls for Annexin V-FITC and PI staining in IHEEC and IHESC used for gating. (D) Comparison of cell viability between cells treated with 3.5 µL of Lipofectamine RNAiMAX (control) and cells treated with 7.5 µL of Lipofectamine RNAiMAX (siControl) in IHEEC. Statistical significance was determined using the Mann–Whitney *U*-test. *P < .05. Abbreviations: FITC, fluorescein isothiocyanate; IHEEC, immortalized human endometriotic epithelial cells; IHESC, immortalized human endometriotic stromal cells; PI, propidium iodide.

Apoptotic cells and cell viability following receptor knockdown were assessed by detecting phosphatidylserine using an Annexin V-fluorescein isothiocyanate apoptosis detection kit (V13241, Thermo Fisher Scientific) according to the manufacturer's protocol. IHEEC and IHESC cells were seeded in 6-well plates and transfected with siRNAs for 72 hours. After transfection, cells were washed with ice-cold phosphate-buffered saline (PBS), then stained with 2 µL Alexa Fluor 488–conjugated Annexin V and 100 µg/mL propidium iodide (PI) for 15 minutes at room temperature. Data acquisition was performed using the Cytek® Aurora flow cytometer (Cytek, Fremont, CA, USA) as shown in Figs. 1A to 1C, and analysis was conducted with FlowJo software (Tree Star Inc., Ashland, OR, USA) to determine cell viability (Annexin V and PI negative), apoptosis (Annexin V positive and PI negative), and apoptotic/necrotic states (Annexin V and PI positive). Cell viability was decreased by siControl digonucleotide (Fig. 1C).

### Immunofluorescence

IHEEC and IHESC cells were seeded in a Nunc™ Lab-Tek™ II Chamber Slide™ System (#154526, Thermo Fisher Scientific) followed by DIM-3,5 analogs at 13 µM for 12 hours. The cells were fixed with 4% paraformaldehyde in PBS for 20 minutes at room temperature. Cells were then rinsed with PBS and blocked in 10% horse serum in 0.05% Triton in PBS for 1 hour at room temperature. After blocking, cells were incubated with primary antibody against TWIST1, COL1A1, and N-cadherin in the antibody dilution buffer for 1 hour at room temperature. Following several PBS washes, cells were incubated with fluorochrome-conjugated secondary antibody diluted in antibody dilution buffer for 1 hour at room temperature. Phalloidin (#8953S, Cell Signaling Technology) was then added and incubated for 15 minutes to visualize filamentous actin, followed by Hoechst (#62249, Thermo Fisher Scientific) staining for 1 minute to label cell nuclei. Finally, the slides were imaged using a ZEISS Axio Imager M2, and analysis was conducted with ZEISS ZEN lite software (RRID:SCR_013672).

### Animal Numbers and Power Calculations

The required minimal number of animals per group was determined using a power calculation to ensure adequate statistical power (α = .05, power = 80%) to detect a biologically significant difference. Sample size estimation was performed using GPower (40) based on each experiment’s data.

### Mice

FVB/NJ female mice (6 weeks old) were purchased from Jackson Laboratory. FVB mice were maintained in the designated animal care facility at Baylor College of Medicine according to the IACUC guidelines for the care and use of laboratory animals. An IACUC-approved protocol was followed for all animal experiments in this study. The assurance number of our animal protocol is D16-00475.

### Immortalized Human Endometrial Cells

Using our primary human endometrial stromal cells isolated from an ovarian endometrioma (31), we generated immortalized human endometriotic stromal cells using lentivirus expressing human telomerase reverse transcriptase (31). EMosis-CC/TERT1 (immortalized human endometriotic epithelial cells) (41) were employed. Cells were confirmed by short tandem repeat profiling; these cells were not contaminated with mycoplasma.

### Induction of Endometriosis in Mice

Luciferase-labeled female mice [FVB-Tg(CAG-luc, -GFP)L2G85Chco/J, 6 weeks old] were used as uterine tissue donors, and syngeneic FVB/NJ female mice (6 weeks old) served as recipients. Uterine horns were harvested from donor mice. The isolated uterine horns were longitudinally opened using surgical scissors. Uterine tissue discs were then prepared using a 2-mm dermal biopsy punch (Miltex, Bethpage, NY, USA). These tissue discs were sutured to the peritoneal wall or mesenteric membrane of the same mouse through the midline incision using a 7-0 Vicryl suturing kit (synthetic absorbable material). Finally, the abdominal wall and skin were closed separately. The abdominal wall was sutured with 7-0 PDS in a continuous fashion, followed by separate closure of the skin.

### Determine Effect of DIM-3,5-Cl_2_ on Endometriosis Progression With Mice

Endometriosis was surgically induced in FVB/NJ female mice (6 weeks old, n = 10) as previously described. Two weeks postsurgery, when endometriotic lesions were established, mice were randomly assigned to 2 groups. One group (n = 5) received daily intraperitoneal injections of DIM-3,5-Cl_2_ (2.5 mg/kg) for 34 consecutive days. The control group (n = 5) received daily intraperitoneal injections of vehicle (5% DMSO and 10% 2-hydroxypropyl-β-cyclodextrin) for the same duration. Luciferase activity of ectopic lesions was measured weekly using an in vivo imaging system. After the final imaging session, the sizes of ectopic lesions were measured to assess treatment effects.

### Liver Panel Assay

After final image analysis, whole blood was collected and allowed to clot by leaving it at room temperature for 20 minutes. After centrifuging the clot at 1000 to 2000 × g for 10 minutes in a refrigerated centrifuge, supernatant serum was collected. In the liver panel assay, levels of total protein, alanine aminotransferase, aspartate aminotransferase, alkaline phosphatase, total-value bilirubin, direct bilirubin, indirect bilirubin, and albumin–globulin ratio in serum were determined by the Clinical Pathology Core in the Center for Comparative Medicine in Baylor College of Medicine.

### Tissue Processing and Immunohistochemistry

Mouse ectopic lesions were harvested, fixed in 10% neutral-buffered formalin, and paraffin-embedded following routine tissue processing. The paraffin-embedded tissues were sectioned at a thickness of 7 μm. The sections were deparaffinized in xylene, rehydrated through a graded ethanol series, and then subjected to immunostaining. Antigen retrieval was performed using a citrate-based buffer (pH 6.0; # H-3300) from Vector Laboratories (Newark, CA, USA). Specific antigens were visualized using a DAB substrate kit (#SK-4100, RRID:AB_2336382) from Vector Laboratories. H-scores of the stained images were quantified using QuPath software (42).

### Terminal Deoxynucleotidyl Transferase Biotin-dUTP Nick End Labeling Assay

The FFPE sections were deparaffinized in xylene, rehydrated through a graded ethanol series, and subjected to terminal deoxynucleotidyl transferase biotin-dUTP nick end labeling (TUNEL) assay. The ApopTag Fluorescein In Situ Apoptosis Detection Kit (#S7110, Millipore Sigma) was used according to the manufacturer's instructions. Nuclei were counterstained with Hoechst 33342 (#B2883, Millipore Sigma). Stained cells were imaged using a Keyence BZ-X800 microscope. Between 6 and 12 random fields of view were captured and analyzed for the percentage of TUNEL-positive cells using QuPath software (42).

### Statistical Analysis

All the experiments were repeated a minimum of 3 times. The data are expressed as the mean ± SE/SD. One-way analysis of variance and the Mann–Whitney *U*-test was used to determine statistical significance. *P*-values < .05 were considered statistically significant.

## Results

### NR4A1 and NR4A2 Have an Essential Role in Cell Viability of IHEECs and IHESCs

**Figure 2.**
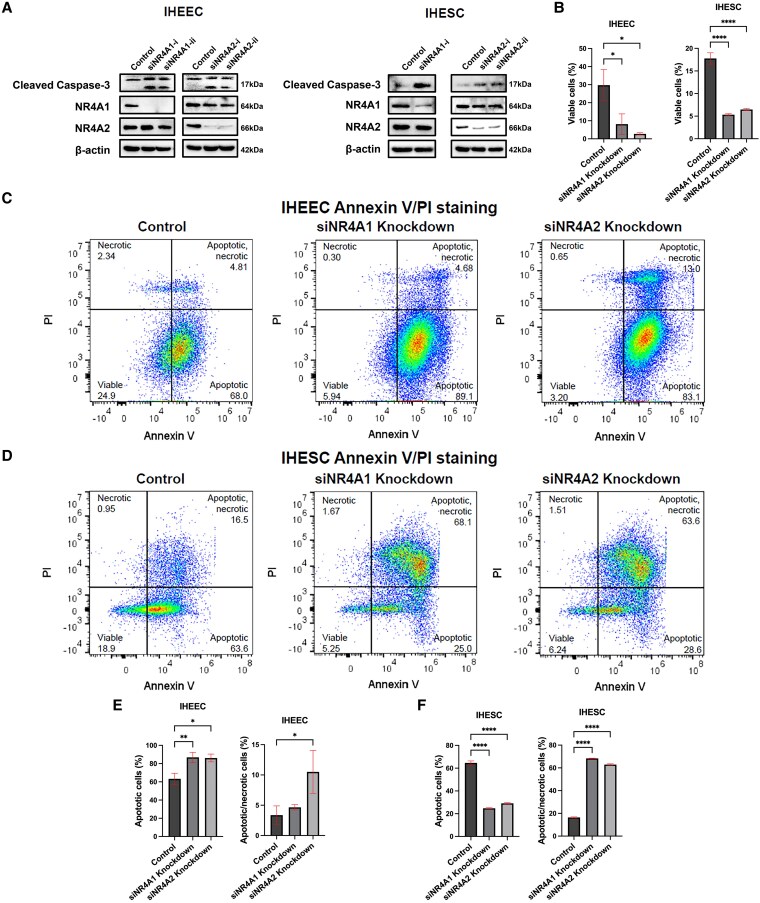
Roles of NR4A1 and NR4A2 in endometriotic cell growth and apoptosis. IHEEC and IHESC cells were transfected with siNR4A1 and siNR4A2, and effects on protein levels (A) and cell viability (B) were determined as outlined in the Methods. Apoptosis was analyzed 72 hours after transfection of IHEEC and IHESC cells with siRNAs targeting NR4A1 (siNR4A1) or NR4A2 (siNR4A2). Representative flow cytometry dot plots showing apoptosis profiles in IHEEC and IHESC are presented (C, D). In each plot: the upper left quadrant (Q1) represents necrotic cells (PI-positive only), the upper right quadrant (Q2) shows late apoptotic cells (Annexin V-FITC and PI double-positive), the lower right quadrant (Q3) indicates early apoptotic cells (Annexin V-FITC–positive only), and the lower left quadrant (Q4) represents viable cells (double-negative). Quantification of early apoptotic (E) and late apoptotic (F) cell populations is shown. Statistical significance was determined as follows: *P < .05, **P < .01, ***P < .001. Abbreviations: FITC, fluorescein isothiocyanate; IHEEC, immortalized human endometriotic epithelial cells; IHESC, immortalized human endometriotic stromal cells; NR4A1, nuclear receptor 4A1; NR4A2, nuclear receptor 4A2; PI, propidium iodide; siRNA, small interfering RNA.

Both NR4A1 and NR4A2 are expressed in endometriotic lesions (31, 32). While NR4A1 plays an essential role in human endometriotic cells (31, 32), the function of NR4A2 in human endometrial cells has not been previously investigated. To address this gap, we determined the effects of NR4A1 and NR4A2 knockdown (KD) by RNA interference on the viability and survival of IHEECs and IHESCs using flow cytometry, with Annexin V and PI staining to assess cell death. siRNAs targeting NR4A1 or NR4A2 effectively reduced levels only of their respective proteins compared to nontargeting siRNA controls (Fig. 2A) confirming knockdown specificity (Fig. 2A). Furthermore, siRNA-mediated KD of NR4A1 or NR4A2 triggers apoptotic signaling, as indicated by increased levels of cleaved caspase-3 (Fig. 2A). Viability assays revealed that KD of either NR4A1 or NR4A2 significantly decreased the viability of IHEECs and IHESCs to a similar extent (Fig. 2B) indicating that NR4A2 plays a similar role compared to NR4A1 in maintaining the viability of human endometriotic epithelial and stromal cells. Notably, overall cell viability was low in both KD and control groups after 72 hours of transfection (Fig. 1D), which is at least partly attributable to transfection reagent toxicity.

In IHEECs, both NR4A1 and NR4A2 KD significantly increased apoptotic cells (Annexin V⁺ PI⁻), which led to a significant increase in apoptotic/necrotic cells (Annexin V⁺ PI⁺) compared to control KD (Fig. 2C and 2E). In contrast, in IHESCs, KD of both NR4A1 and NR4A2 predominantly and significantly elevated the proportion of apoptotic/necrotic cells (Annexin V⁺ PI⁺) (Fig. 2D and 2F). These results suggest that both NR4A1 and NR4A2 are required for survival of endometriotic stromal and epithelial cells, with knockdown of either inducing cell death, particularly in stromal cells, potentially triggering local inflammation.

### DIM-3,5 Derivatives Effectively Suppressed the Growth of Human Endometriotic Cell

**Figure 3.**
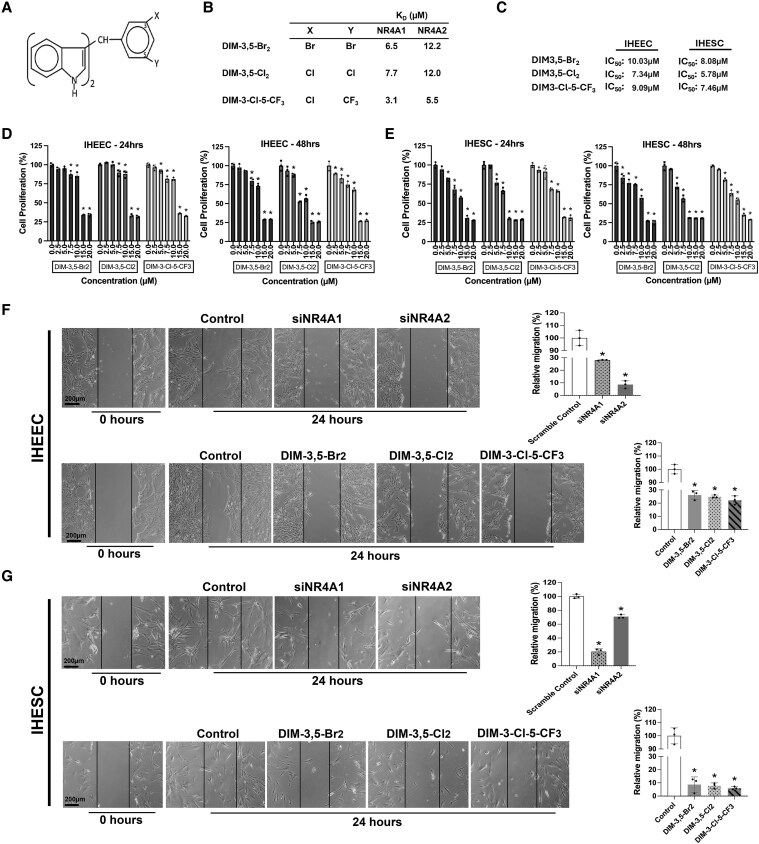
Effects of DIM-3,5 analogs on proliferation and migration of IHEEC and IHESC cells. The structure of the DIM-3,5 analogs (A) and corresponding dissociation constants (Kd) values of selected DIM-3,5 analogs for NR4A1 and NR4A2 are shown (B). IC_50_ values for IHEEC and IHESC cells at 48 hours, determined from XTT assays (C). IHEEC and stromal IHESC cells were treated with selected DIM-3,5 analogs, and cell proliferation was assessed using XTT assays, as described in the Methods section (D, E). IHEEC (F) and IHESC (G) cells were transfected with oligonucleotides targeting NR4A1 or NR4A2 or treated with DIM-3,5 analogs (6.5 µM) for 24 hours, and effects on cell migration were determined in a scratch assay as outlined in the Materials and Methods. Data are presented as means ± SD for 3 replicate determinations, and statistical differences (*P* < .05) of treated vs controls (DMSO) are indicated (*). Abbreviations: DIM-3,5, 1,1-bis(3′-indolyl)-(3,5-disubstitutedphenyl)methane; DMSO, dimethyl sulfoxide; IHEEC, immortalized human endometriotic epithelial cells; IHESC, immortalized human endometriotic stromal cells; NR4A1, nuclear receptor 4A1; NR4A2, nuclear receptor 4A2.

DIM-3,5 derivatives were recently characterized as dual NR4A1/NR4A2 ligands that bind to both NR4A1 and NR4A2 (38). They exhibited potent inverse agonist activity in cancer cells and inhibited the expression of NR4A1- and NR4A2-regulated pro-oncogenic pathways (37, 39). Among the compounds, DIM-3,5-Br_2_, DIM-3,5-Cl_2_, and DIM-3-Cl-5-CF_3_ were selected for the endometriosis study based on their strong inverse agonist activity and favorable pharmacological properties (Fig. 3A). These compounds effectively suppressed the proliferation of IHEECs and IHESCs in a dose-dependent manner over a narrow range of concentrations (Fig. 3B and 3C). The IC_50_ values calculated from treatment of IHEEC cells with DIM-3,5-Br_2_, DIM-3,5-Cl_2_, and DIM-3-Cl-5-CF_3_ for 48 hours were 10 μM, 7.34 μM, and 9.09 μM, respectively. Similarly, the IC_50_ values in IHESCs were also 8.08 μM, 5.78 μM, and 7.46 μM, respectively. In addition, we also showed that knockdown of NR4A1 or NR4A2 or treatment with DIM-3,5 analogs decreased migration of IHEEC (Fig. 3D) and IHESC (Fig. 3E) cells. This indicates that both receptors are promigratory and the DIM-3,5 ligands are acting as inverse agonists to inhibit migration of endometriotic cells.

### DIM-3,5 Derivatives Suppressed EGFR Levels, Increasing Cell-death Signaling in Human Endometriotic Cells by Inhibiting NR4A1 and NR4A2

**Figure 4.**
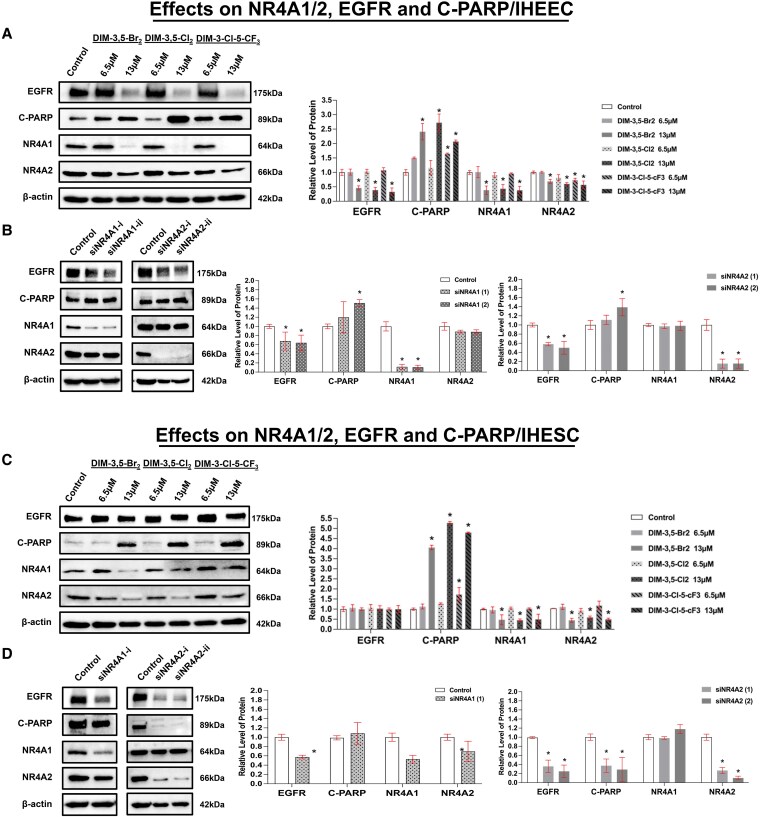
Effects of DIM-3,5 analogs and NR4A knockdown on EGFR and C-PARP expression in IHEEC and IHESC cells. IHEEC cells were treated for 24 hours with DMSO (vehicle control) or selected DIM-3,5 analogs (DIM-3,5-Br_2_, DIM-3,5-Cl_2_, and DIM-3-Cl-5-CF_3_) (A) and oligonucleotides that target NR4A1 and NR4A2 (B). Whole-cell lysates were collected and analyzed by Western blotting, as described in the Methods section. IHESC cells were also treated with DIM-3,5 ligands (C) and transfected with siNR4A1 or siNR4A2 (D), and whole-cell lysates were obtained and analyzed by Western blots as outlined in the Methods. Protein expression levels were quantified by measuring relative band intensities compared to DMSO controls (set at 1.0) and normalized to β-actin (A-D). The same lysates from IHEEC cells were used for the blots shown in Figs. 3A and 4A, and the same lysates from IHESC cells were used for the blots shown in Figs. 3D and 5D; therefore, the β-actin loading controls are identical. Data are presented as mean ± SD. *P < .05. Abbreviations: C-PARP, cleaved poly(ADP-ribose) polymerase; DIM-3,5, 1,1-bis(3′-indolyl)-(3,5-disubstitutedphenyl)methane; DMSO, dimethyl sulfoxide; EGFR, epidermal growth factor receptor; IHEEC, immortalized human endometriotic epithelial cells; IHESC, immortalized human endometriotic stromal cells; NR4A1, nuclear receptor 4A1; NR4A2, nuclear receptor 4A2.

**Figure 5.**
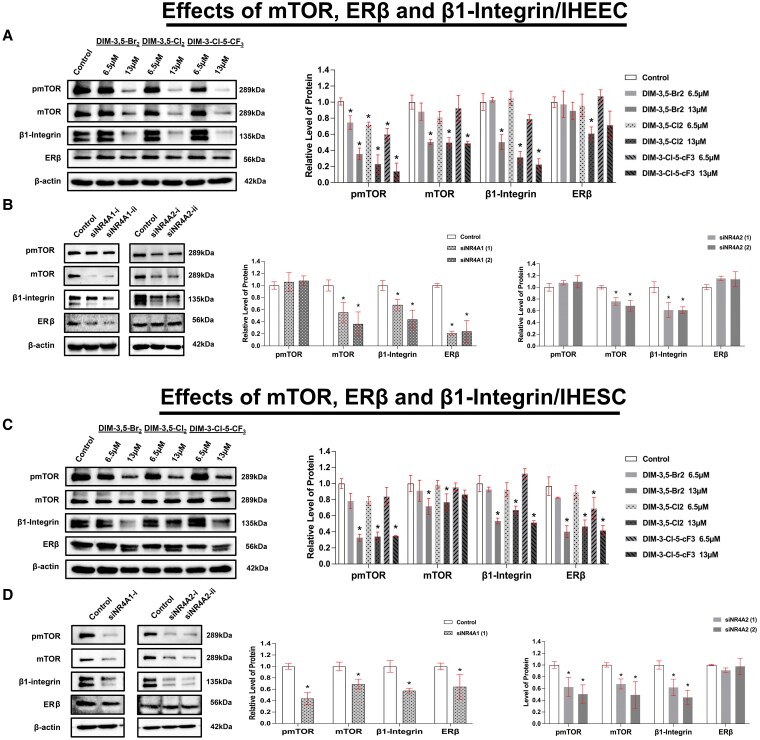
Effects of DIM-3,5 analogs NR4A1 and NR4A2 knockdown on β1-integrin, estrogen receptor β, and mTOR signaling in IHEEC and IHESC cells. IHEEC cells were treated with DIM-3,5 analogs for 24 hours (A) and transfected with siRNAs targeting NR4A1 (siNR4A1) or NR4A2 (siNR4A2) (B) for 72 hours. Whole-cell lysates were then collected and analyzed by Western blotting, as described in the Methods section. IHESC cells were treated with DIM-3,5 analogs for 24 hours (C) and transfected with siNR4A1 or siNR4A2 for 72 hours (D). Whole-cell lysates were analyzed by Western blots. Protein expression levels were quantified by measuring relative band intensities compared to control siRNA-treated cells (set at 1.0) and normalized to β-actin (A–D). Data are presented as mean ± SD. *P < .05. Abbreviations: DIM-3,5, 1,1-bis(3′-indolyl)-(3,5-disubstitutedphenyl)methane; IHEEC, immortalized human endometriotic epithelial cells; IHESC, immortalized human endometriotic stromal cells; mTOR, mechanistic target of rapamycin; NR4A1, nuclear receptor 4A1; NR4A2, nuclear receptor 4A2; siRNA, small interfering RNA.

**Figure 6.**
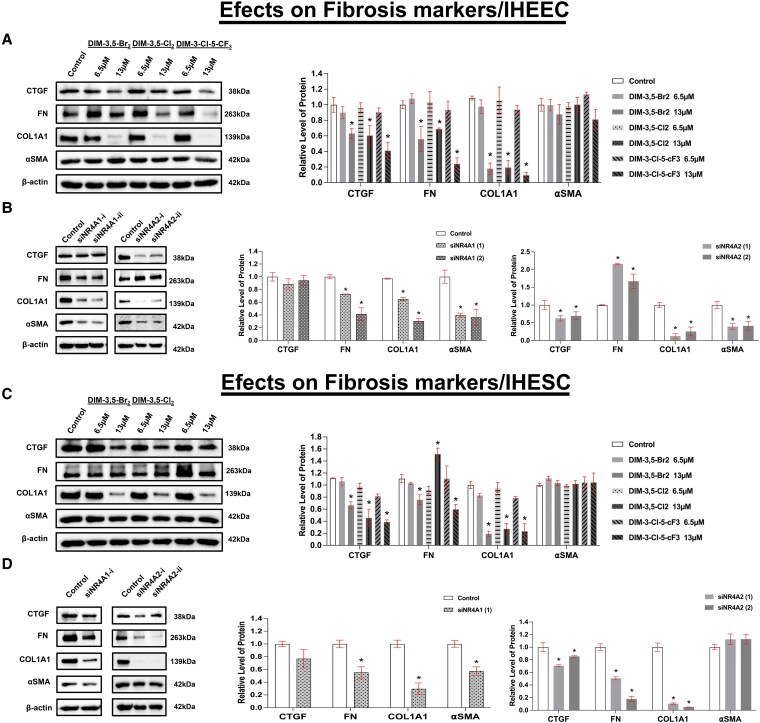
Effects of DIM-3,5 analogs and NR4A1/NR4A2 knockdown on fibrosis markers in IHEEC and IHESC cells. IHEEC cells were treated for 24 hours with DMSO (vehicle control) or selected DIM-3,5 analogs (DIM-3,5-Br_2_, DIM-3,5-Cl_2_, and DIM-3-Cl-5-CF_3_) (A) and transfected with siNR4A1 or siNR4A2 (B). Whole-cell lysates were collected and analyzed by Western blotting, as described in the Methods section. IHESC cells were treated with DIM-3,5 compound for 24 hours (C) and transfected with siNR4A1 or siNR4A2 for 72 hours (D), and whole-cell lysates were analyzed by Western blots. Protein expression levels were quantified by measuring relative band intensities compared to DMSO controls (set at 1.0) and normalized to β-actin (A-D). The same lysates from IHESC cells were used for the blots shown in Figs. 5B and 6B, as well as Figs. 5C and 6C; therefore, the β-actin loading controls are identical. Data are presented as mean ± SD. *P < .05. Abbreviations: DIM-3,5, 1,1-bis(3′-indolyl)-(3,5-disubstitutedphenyl)methane; DMSO, dimethyl sulfoxide; IHEEC, immortalized human endometriotic epithelial cells; IHESC, immortalized human endometriotic stromal cells; NR4A1, nuclear receptor 4A1; NR4A2, nuclear receptor 4A2.

**Figure 7.**
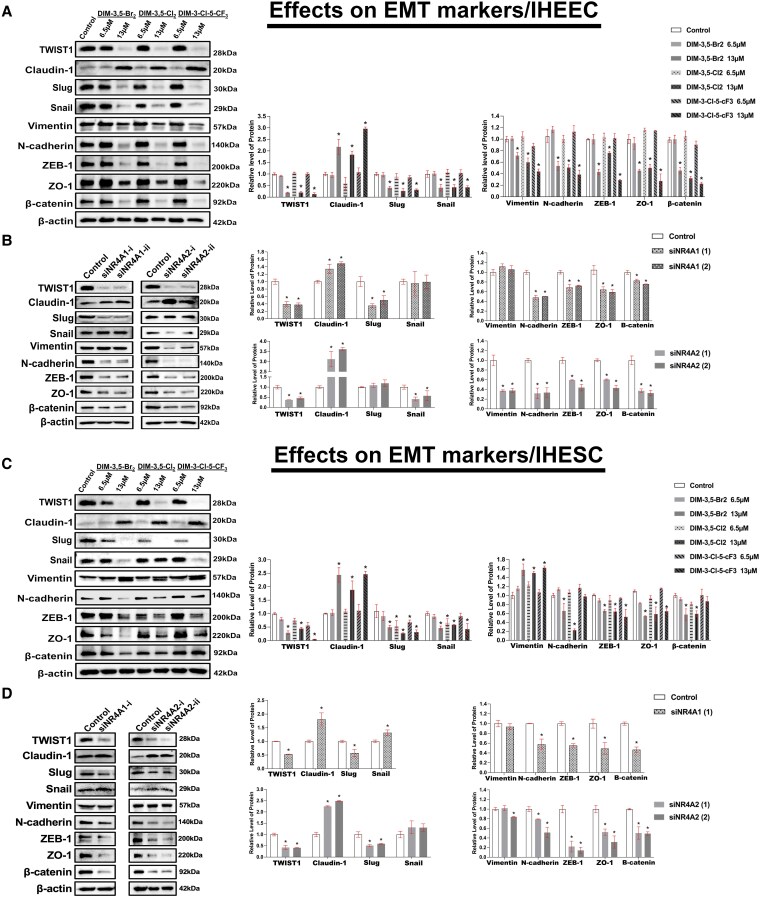
Effects of DIM-3,5 analogs and NR4A1/NR4A2 knockdown on EMT-related markers expression in IHEEC and IHESC cells. IHEEC cells were treated for 24 hours with DMSO (vehicle control) or selected DIM-3,5 analogs (DIM-3,5-Br_2_, DIM-3,5-Cl_2_, and DIM-3-Cl-5-CF_3_) (A) for 24 hours and transfected with siNR4A1 or siNR4A2 (B) for 72 hours. Whole-cell lysates were collected and analyzed by Western blotting, as described in the Methods section. IHESC cells were treated with DIM-3,5 analogs (C) for 24 hours, and transfected with siNR4A1 or siNR4A2 (D) for 72 hours, and whole-cell lysates were analyzed by Western blots. Protein expression levels of EMT markers were quantified by measuring relative band intensities compared to DMSO controls (set at 1.0) and normalized to β-actin (A-D). Data are presented as mean ± SD. *P < .05. Abbreviations: DIM-3,5, 1,1-bis(3′-indolyl)-(3,5-disubstitutedphenyl)methane; DMSO, dimethyl sulfoxide; EMT, epithelial-to-mesenchymal transition; IHEEC, immortalized human endometriotic epithelial cells; IHESC, immortalized human endometriotic stromal cells.

Results in Figs. 2 and 3 indicate that both NR4A1 and NR4A2 exhibit functional proendometriotic activities in IHEEC and IHESC cells. The effects of DIM-3,5 ligands and KD of NR4A1 and NR4A2 on the expression of key endometriosis-related gene products are summarized in Figs. 4 to 7. A comparison between proteins induced or repressed by DIM-3,5 and those effects by receptor KD will be used to determine the role of NR4A1, NR4A2, and NR4A1/NR4A2 (combined) in the regulation of individual proteins. It is possible that DIM-3,5 ligands may exhibit activity as selective receptor modulators and not affect the expression of proteins that are downregulated following receptor KD. This is commonly observed for other nuclear receptor ligands (eg, selective estrogen receptor modulators). Receptor-independent gene products are those induced/repressed by DIM-3,5 ligands and not by receptor KD. In addition, the results show that regulation of genes by DIM-3,5 ligand or receptor KD is also dependent on cell context, varying between IHEEC and IHESC. EGFR signaling has a critical role in endometriosis progression by enhancing cell migration, invasion, angiogenesis, and immune-related pathways (43-45). Previous studies show that NR4A1 ligands downregulated expression of EGFR and induced PARP cleavage (apoptosis) in endometriotic cells (31, 32), and results in Fig. 4A and 4B show that DIM-3,5 compounds (6.5 and 13 µM) and KD of both receptors significantly decreased EGFR protein levels in IHEEC cells compared to the vehicle control (Fig. 4A and 4B). Additionally, C-PARP levels were significantly elevated in IHEECs following treatment with all 3 DIM-3,5 derivatives and by KD of NR4A1 and NR4A2 (Fig. 4A and 4B). Thus both EGFR and C-PARP are coregulated by NR4A1 and NR4A2 in IHEEC cells. In contrast, effects of DIM-3,5 compounds and receptor KD did not correlate for either EGFR or C-PARP in IHESC cells (Fig. 4C and 4D). In IHESC cells, KD of NR4A1 and NR4A2 decreased EGFR, but DIM-3,5 ligands had no effect, whereas C-PARP was induced by DIM-3,5 analogs but not by receptor KD. This suggests that for EGFR, the DIM-3,5 compounds may be acting as a selective receptor modulator that does not activate this specific gene, whereas induction of C-PARP by the ligands is receptor-independent.

### DIM-3,5 Derivatives Suppressed β1-integrin/mTOR/ERβ Signaling Axis in Human Endometriotic Cells by Inhibiting NR4A1 and NR4A2

β1-integrin/mTOR signaling plays a critical role in the progression of endometriosis, and NR4A1 has been shown to modulate this axis to promote disease progression (32). DIM-3,5 compounds decreased mTOR/pmTOR in IHEEC cells, whereas KD of NR4A1 and NR4A2 decreased expression of mTOR but not phospho mTOR (Fig. 5A and 5B), and the reasons for these differences are not apparent and are being further investigated. In contrast, results in IHESC cells show that DIM-3,5 and KD of both receptors decrease mTOR and phospho mTOR (Fig. 5C and 5D). A comparison of the effects of DIM-3,5 and receptor KD on β1-integrin expression shows that β1-integrin is coregulated by NR4A1 and NR4A2 and is downregulated by DIM-3,5 ligands in IHEEC and IHESC cells. ERβ, a major driver of endometriosis (46, 47), is decreased after treatment with DIM-3,5 analogs in IHESC but not IHEEC cells, and results of KD studies show that ERβ is an NR4A1-regulated gene in the former cell line.

### DIM-3,5 Derivatives Inhibited Fibrosis Progression in Human Endometriotic Cells by Inhibiting NR4A1 and NR4A2

NR4A1 is involved in regulating gene expression related to endometriosis-associated fibrosis, thereby contributing to disease progression (31, 32). Therefore, we investigated whether DIM-3,5 derivatives also suppress endometriosis-associated genes linked to fibrosis. In this study, we analyzed the effects of DIM-3,5 ligands and KD of NR4A1 and NR4A2 on CTGF, FN, COL1A1, and α-SMA expression in IHEEC (Fig. 6A and 6B) and IHESC (Fig. 6C and 6D) cells. DIM-3,5 compounds decreased expression of CTGF and COL1A1 in both cell lines, and results of receptor KD studies indicated that CTGF is an NR4A2-regulated gene and COL1A1 is coregulated by both receptors in IHEEC and IHESC cells. DIM-3,5 compound decreases FN in IHEEC but not IHESC cells and is regulated by NR4A1 in IHEEC cells. Interestingly, knockdown of NR4A1 or NR4A2 decreased α-SMA in IHEEC but DIM-3,5 has no effect, suggesting that the compound is acting as a selective receptor modulator for this gene. In contrast, minimal effects on α-SMA expression are observed in IHESC cells after treatment with DIM-3,5 or KD of NR4A1 (slight decrease) or NR4A2 (Fig. 6D).

### DIM-3,5 Derivatives Blocked EMT Progression in Human Endometriotic Cells by Inhibiting NR4A1 and NR4A2

EMT plays a critical role in endometriosis progression, and EMT-related genes are significantly upregulated in patients with endometriosis, promoting enhanced cell migration and invasion (48-51). Interestingly, the expression of TWIST1, a major regulator of EMT, is coregulated by both NR4A1 and NR4A2 in glioblastoma cells (37), and treatment with DIM-3,5 compounds or receptor KD also decreased TWIST1 expression in IHEEC (Fig. 7A and 7B) and IHESC (Fig. 7C and 7D) cells. DIM-3,5 ligands and KD of NR4A1 and NR4A2 downregulated expression of multiple mesenchymal genes and also induced expression of claudin-1, an epithelial marker gene. EMT-related proteins downregulated by DIM-3,5 ligands and KD of both NR4A1 and NR4A2 in IHEEC and IHESC cells include TWIST1, N-cadherin, ZEB1, ZO-1, and β-catenin. DIM-3,5 ligands also decrease the expression of Slug and Snail in IHEEC and IHESC cells, with Slug regulation being primarily NR4A1-dependent. Regulation of Snail in IHEEC cells is NR4A2-dependent, whereas in IHESC cells, DIM-3,5-dependent downregulation is independent of both receptors. DIM-3,5-mediated downregulation of Vimentin in IHEEC cells was NR4A2-dependent, whereas the induction of Vimentin by DIM-3,5 analogs was receptor-independent. These results show that most of the EMT markers induced or repressed by DIM-3,5 ligands were coregulated by NR4A1 and NR4A2.

**Figure 8.**
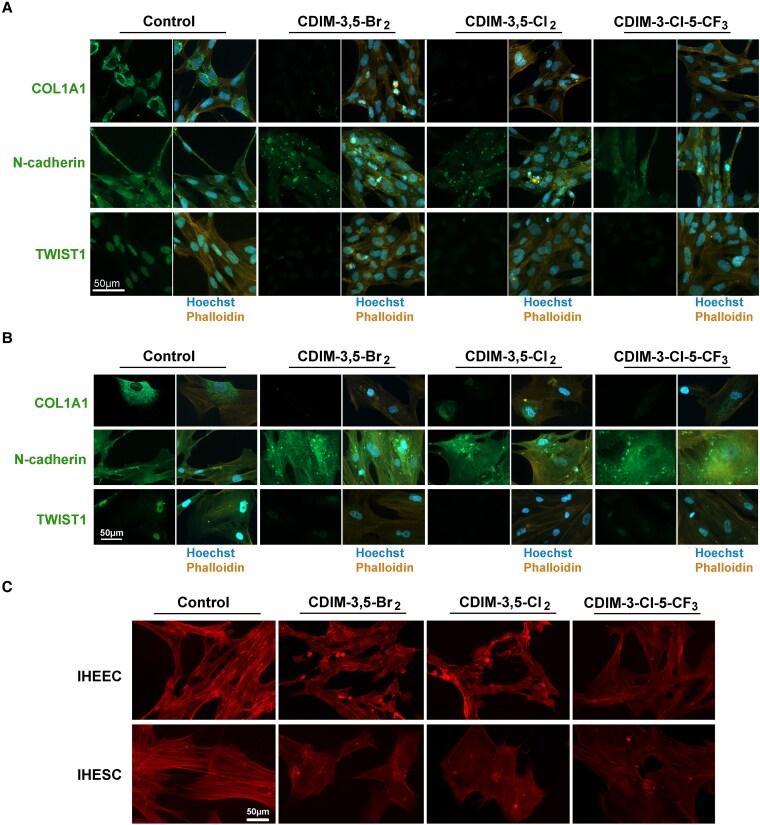
Immunofluorescence staining of fibrotic and EMT markers in IHEEC and IHESC cells. IHEEC (A) and IHESC (B) cells were treated with 13 μM of selected DIM-3,5 analogs (DIM-3,5-Br_2_, DIM-3,5-Cl_2_, and DIM-3-Cl-5-CF_3_) for 12 hours. Immunofluorescence staining was performed for COL1A1, N-cadherin, and TWIST1, all visualized in green, as outlined in the Methods section. Nuclei were counterstained with Hoechst (blue), and actin filaments were visualized with phalloidin (yellow). (C) Representative images showing the effects of DIM-3,5 treatment on actin filament organization. Scale bar = 50 μm. Abbreviations: DIM-3,5, 1,1-bis(3′-indolyl)-(3,5-disubstitutedphenyl)methane; EMT, epithelial-to-mesenchymal transition; IHEEC, immortalized human endometriotic epithelial cells; IHESC, immortalized human endometriotic stromal cells.

To validate the effects of DIM-3,5 derivatives on human endometriotic cells as determined by Western blot analysis, immunofluorescence staining was performed to assess changes in the expression of fibrotic and EMT markers in both human endometriotic epithelial and stromal cells (Fig. 8). Cells were stained with antibodies against COL1A1, N-cadherin, and TWIST1. Cell nuclei and filamentous actin were visualized using Hoechst and fluorescently labeled phalloidin, respectively. DIM-3,5 treatment markedly reduced the fluorescence intensity of COL1A1, N-cadherin, and TWIST1 compared to untreated controls in both epithelial (Fig. 8A) and stromal cells (Fig. 8B). These immunofluorescence results strongly support the regulatory effects of DIM-3,5 derivatives on fibrosis- and EMT-associated pathways observed in Western blot analysis. Additionally, phalloidin staining revealed a substantial decrease in filamentous actin structures in treated cells (Fig. 8C), suggesting that DIM-3,5 disrupts actin filament formation or stability.

### DIM-3,5-Cl_2_ Suppressed Endometriosis Progression in Mice Without Cytotoxicity

**Figure 9.**
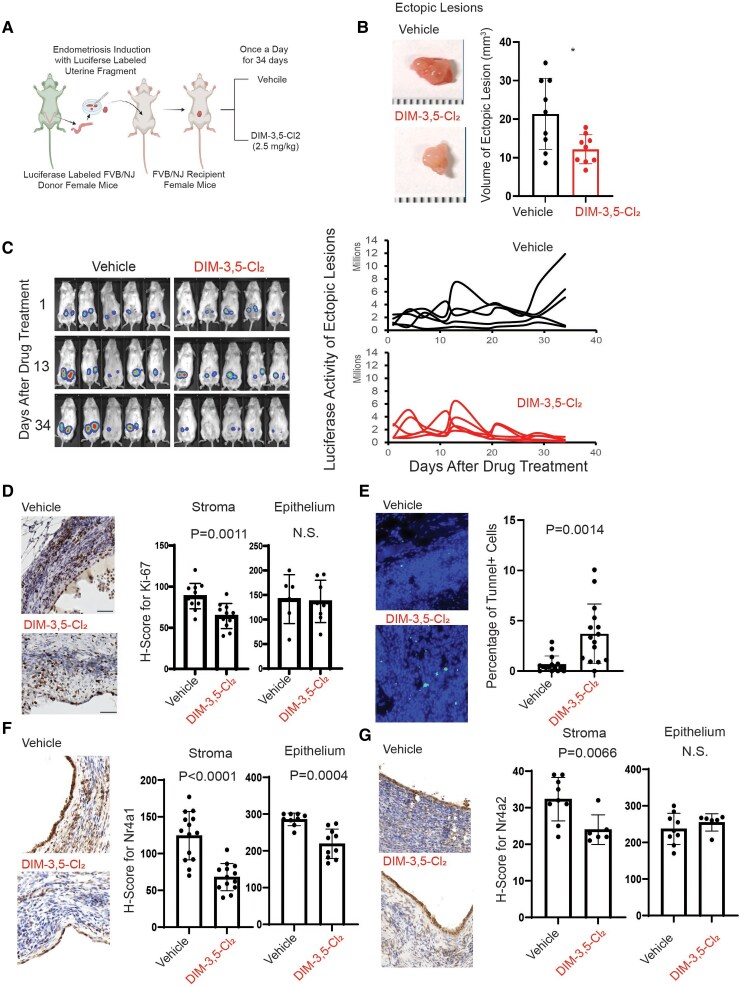
Suppression of ectopic lesion growth in a mouse model of endometriosis by DIM-3,5-Cl_2_ treatment. Mice with surgically induced endometriosis were treated once daily with DIM-3,5-Cl_2_ (2.5 mg/kg) or vehicle control for 34 days (A). Following treatment, ectopic lesions were harvested, and their volumes were calculated using the formula: 0.5 × Length × Width^2^ (B). Luciferase activity of ectopic lesions was monitored throughout the treatment period using an in vivo imaging system. Luciferase signal intensity for each lesion was quantified and plotted in the corresponding graph (C). Proliferation in ectopic lesions was assessed by Ki-67 IHC (D). The levels of NR4a1 and Nr4a2 in epithelial and stromal compartments were determined by IHC (F, G), and H-scores were calculated using QuPath software (42). Apoptosis was evaluated by TUNEL assay (E), and the percentage of TUNEL-positive cells in ectopic lesions was quantified using QuPath. H-scores for Nr4a1 and Nr4a2 expression in each cell compartment were also determined using QuPath. Abbreviations: DIM-3,5, 1,1-bis(3′-indolyl)-(3,5-disubstitutedphenyl)methane; IHC, immunohistochemistry; N.S., not significant; TUNEL, terminal deoxynucleotidyl transferase biotin-dUTP nick end labeling.

**Figure 10.**
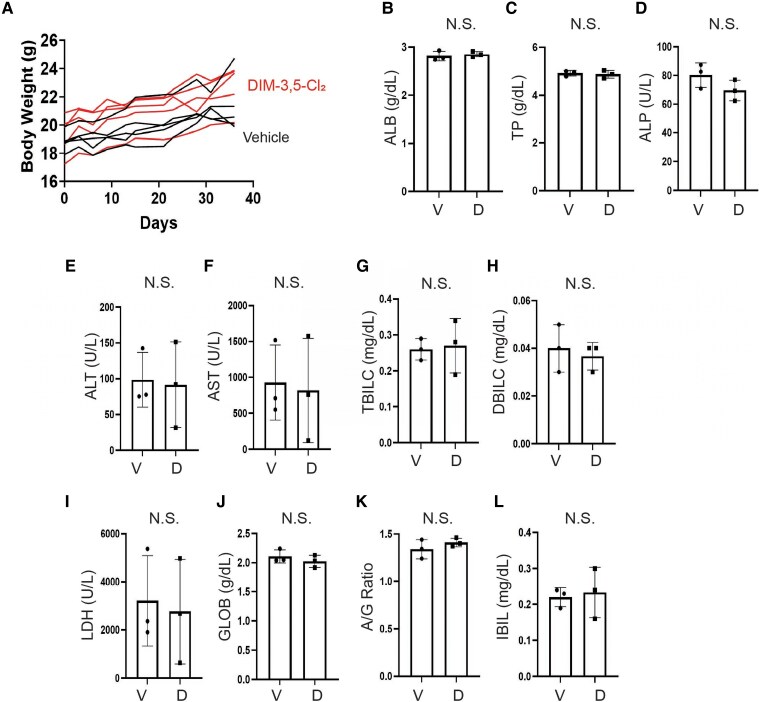
No toxicity of DIM-3,5-Cl_2_ in mice with endometriosis. Body weight changes in mice with endometriosis were monitored 3 times per week during treatment with DIM-3,5-Cl_2_ or vehicle (A). Following the final treatment, whole blood was collected for biochemical analysis. Levels of liver metabolic enzymes and metabolites, including albumin (B), total protein (C), alkaline phosphatase (D), alanine aminotransferase (E), aspartate aminotransferase (F), total bilirubin (G), direct bilirubin (H), lactate dehydrogenase (I), globulin (J), albumin/globulin ratio (K), and indirect bilirubin (L), were measured in mice treated with DIM-3,5-Cl_2_ or vehicle. *P* < .05. Abbreviations: D, DIM-3,5-Cl_2_; N.S., not significant; V, vehicle.

All in vitro data clearly demonstrated that DIM-3,5 derivatives inhibited key cellular pathways and genes that drive endometriosis in human endometriotic epithelial and stromal cells, and these effects were dependent on 1 or both receptors (coregulation). Next, we assessed whether DIM-3,5 derivatives could effectively suppress endometriosis progression in vivo using an endometriosis-induced mouse model. To noninvasively monitor the growth of ectopic lesions, we utilized a luciferase-expressing mouse line that expresses luciferase in all tissues (31, 32). Uterine tissues were isolated from luciferase-labeled donor mice, and fragmented uterine pieces were attached to the peritoneal membrane of syngeneic female recipient mice (FVB/NJ) to induce endometriosis (Fig. 9A). Mice with established endometriosis were treated with DIM-3,5-Cl_2_ (2.5 mg/kg/day) or vehicle control for 34 days (Fig. 9A). At the end of the treatment period, ectopic lesions were harvested. DIM-3,5-Cl_2_ treatment significantly reduced ectopic lesion volume compared to the vehicle-treated group (Fig. 9B). Using luciferase-based imaging, we noninvasively monitored the sequential growth of ectopic lesions in mice with endometriosis following DIM-3,5-Cl_2_ treatment compared to vehicle controls. Luciferase imaging revealed a continuous increase in ectopic lesion growth in the vehicle-treated group (Fig. 9C). In contrast to the vehicle, DIM-3,5-Cl_2_ significantly inhibited the growth of ectopic lesions in mice with endometriosis (Fig. 9C). To validate the growth-suppressive activity of DIM-3,5-Cl_2_ on ectopic lesions, we assessed cellular proliferation and cell death signaling within the lesions. Immunohistochemistry using a Ki-67 antibody revealed that DIM-3,5-Cl_2_ treatment significantly reduced Ki-67 expression in stromal cells of ectopic lesions compared to the vehicle group (Fig. 9D). However, Ki-67 expression in epithelial cells was not significantly affected by DIM-3,5-Cl_2_ treatment (Fig. 9D). The TUNEL assay also showed that DIM-3,5-Cl_2_ treatment significantly increased the number of TUNEL-positive cells in ectopic lesions compared to the vehicle group (Fig. 9E). Together, these findings indicate that DIM-3,5-Cl_2_ treatment reduces cellular proliferation and enhances cell death signaling in ectopic lesions, thereby suppressing the progression of endometriosis. Our in vitro analysis demonstrated that DIM-3,5-Cl_2_ treatment significantly reduced NR4A1 and NR4A2 levels in human endometrial cells (Fig. 4). To determine whether the endometriosis-suppressive effect of DIM-3,5-Cl_2_ is associated with downregulation of these receptors in vivo, we assessed their expression in ectopic lesions from DIM-3,5-Cl_2_– and vehicle-treated mice. Immunohistochemistry revealed that DIM-3,5-Cl_2_ significantly reduced Nr4a1 expression in both epithelial and stromal cells of ectopic lesions (Fig. 9F). Similarly, Nr4a2 levels were significantly decreased in stromal cells following DIM-3,5-Cl_2_ treatment compared to vehicle controls (Fig. 9F). However, Nr4a2 expression in epithelial cells was not significantly affected (Fig. 9G). These results suggest that the suppressive effect of DIM-3,5-Cl_2_ on endometriosis is associated with the downregulation of NR4A1 and NR4A2 in ectopic lesions. To assess the potential toxicity of DIM-3,5-Cl_2_ during endometriosis treatment, changes in body weight and liver function panels were evaluated. DIM-3,5-Cl_2_ treatment did not result in body weight loss over the treatment period (Fig. 10A). Additionally, a liver panel analysis of blood samples indicated no evidence of liver damage in DIM-3,5-Cl_2_–treated mice compared to controls (Fig. 10B to 10L). These findings suggest that DIM-3,5-Cl_2_ does not induce overt cytotoxicity in vivo during the suppression of endometriosis progression.

Collectively, these complementary in vitro and in vivo results indicate that NR4A1 and NR4A2 are critical proendometriotic genes and that DIM-3,5 derivates effectively target these pathways to suppress endometriosis progression.

## Discussion

Endometriosis significantly impacts patients' quality of life due to symptoms such as chronic pain and infertility. However, current hormone-based therapies fail to effectively halt disease progression or adequately alleviate endometriosis-associated symptoms and often cause severe adverse effects. Therefore, there is a critical need to identify specific cellular pathways closely linked to endometriosis progression to develop alternative, nonhormonal therapies. NR4A1 has emerged as a promising druggable target. The NR4A1 inverse agonist 1,1-bis(3′-indolyl)-1-(3-chloro-4-hydroxy-3-methoxyphenyl) methane (DIM-C-pPhClOH) inhibits endometriotic cell growth, survival, mTOR signaling, and fibrosis (31) and suppresses endometriosis progression in mouse models (31, 32). EndometDB data show that both NR4A1 and NR4A2 are elevated in endometriosis patients compared to healthy controls (52). This suggests that NR4A2 may also be involved in endometriosis progression because NR4A2 has been implicated in regulating cell proliferation, EMT, mTOR signaling, and fibrosis in various tissues and human diseases (53-58). However, the specific role of NR4A2 in endometriosis has not yet been investigated. Here, we demonstrate that NR4A2 plays an essential role in activating endometriosis-driving cellular pathways and gene products in human endometriotic epithelial and stromal cells. Our study introduces a new concept that 2 NR4A isoforms, NR4A1 and NR4A2, play critical roles in endometriosis progression, providing deeper insight into how orphan nuclear receptors contribute to the pathogenesis of this disease.

Endometriotic lesions are composed of both epithelial and stromal cells, and paracrine communication between these 2 cell types plays a critical role in the progression of endometriosis (56). This raises a key question regarding how NR4A1 and NR4A2 are distinctly involved in epithelial vs stromal compartments to drive endometriosis progression. Our comparative Western blot analysis using NR4A1- and NR4A2-KD in human endometriotic epithelial and stromal cells revealed cell type-specific roles of NR4A isoforms. In human endometriotic cells, both NR4A1 and NR4A2 regulate EGFR signaling in IHEEC but not IHESC cells (Fig. 4) where DIM-3,5 did not affect EGFR expression. Notably, only NR4A1 exhibited a functional association with ERβ in epithelial cells whereas DIM-3,5 and receptor KD had no effect on ERβ in IHEEC (Fig. 5). Results of DIM-3,5 treatment or receptor KD showed that the effects on individual fibrotic gene products were somewhat variable; only COL1A1 was coregulated by both NR4A1 and NR4A2 and DIM-3,5 decreased expression of this gene product in both IHEEC and IHESC cells (Fig. 6). In contrast, among the 9 EMT gene products that were downregulated or induced (only claudin-1) by DIM-3,5, 7 were coregulated by NR4A1 and NR4A2 (Fig.6), suggesting loss of mesenchymal characteristics, and may be indicative of a shift toward mesenchymal-to-epithelial transition.

DIM-3,5 derivatives lacking a 4-hydroxy group—such as DIM-3,5-Br_2_, DIM-3,5-Cl_2_, and DIM-3-Cl-5-OCF_3_—also function as dual NR4A1/NR4A2 ligands (38). These compounds demonstrated IC_50_ values of less than 1 mg/kg/day for inhibiting breast tumor growth in a mouse xenograft model, highlighting their potential for repurposing in endometriosis treatment (39). Compared to NR4A1 or NR4A2 KD alone, DIM-3,5 derivatives more effectively suppressed endometriosis-driving cellular pathways in both human endometriotic epithelial and stromal cells, likely due to their ability to act as inverse agonists and simultaneously inhibit both NR4A1 and NR4A2-mediated pathways and genes. Beyond in vitro efficacy, DIM-3,5-Cl_2_ significantly reduced endometriosis progression in a mouse model. Therefore, dual targeting of NR4A1 and NR4A2 using DIM-3,5 derivatives represents a promising nonhormonal therapeutic strategy to replace current hormone-based treatments, which often cause harmful side effects.

The NR4A1 knockout mice are viable and fertile (56), whereas NR4A2 knockout mice are perinatally lethal, rendering them nonviable and infertile as homozygous nulls (57). This lethality highlights the essential role of NR4A2 in early development and homeostasis; however, inhibition of NR4A2 shows some promise as a potential agent for treating multiple injuries (58-60). In this study, we have taken advantage of the newly characterized DIM-3,5 derivatives that act as dual NR4A1/NR4A2 modulators (38). DIM-3,5-mediated inhibition of both receptors results in the simultaneous targeting of both NR4A1 and NR4A2-dependent endometriosis-driving pathways, resulting in an efficient inhibition of endometriosis. Notably, DIM-3,5-Cl_2_ at a dose of 2.5 mg/kg/day effectively suppressed endometriosis progression in mice without inducing toxicity, demonstrating the therapeutic potential of dual NR4A1/NR4A2 inhibition that is accompanied by a high safety profile. Although DIM-3,5 derivatives exhibit low toxicity in mice (34-39), their potential for severe adverse effects in human trials cannot be excluded. To enhance the specificity of DIM-3,5–based therapies, strategies such as endometriotic lesion–specific antibody-drug conjugates (61) or NR4A2-targeting proteolysis-targeting chimeras (62) are also being explored to mitigate off-target effects and improve therapeutic safety.

As dual inhibitors of NR4A1 and NR4A2, DIM-3,5 derivatives represent a promising nonhormonal therapeutic option for endometriosis, offering the potential to improve treatment efficacy while minimizing the adverse effects associated with current hormonal therapies.

## Data Availability

Original data generated and analyzed during this study are included in this published article or in the data repositories listed in References.

## References

[1] Eskenazi B, Warner ML. Epidemiology of endometriosis. Obstet Gynecol Clin North Am. 1997;24(2):235‐258. 9163765 10.1016/s0889-8545(05)70302-8

[2] Buck Louis GM, Hediger ML, Peterson CM, et al Incidence of endometriosis by study population and diagnostic method: the ENDO study. Fertil Steril. 2011;96(2):360‐365. 21719000 10.1016/j.fertnstert.2011.05.087 PMC3143230

[3] Zondervan KT, Becker CM, Koga K, Missmer SA, Taylor RN, Vigano P. Endometriosis. Nat Rev Dis Primers. 2018;4(1):9. 30026507 10.1038/s41572-018-0008-5

[4] Greene AD, Lang SA, Kendziorski JA, Sroga-Rios JM, Herzog TJ, Burns KA. Endometriosis: where are we and where are we going? Reproduction. 2016;152(3):R63‐R78. 27165051 10.1530/REP-16-0052 PMC4958554

[5] Signorile PG, Viceconte R, Baldi A. New insights in pathogenesis of endometriosis. Front Med (Lausanne). 2022;9:879015. 35572957 10.3389/fmed.2022.879015 PMC9095948

[6] Słopień R, Męczekalski B. Aromatase inhibitors in the treatment of endometriosis. Prz Menopauzalny. 2016;1(1):43‐47. 10.5114/pm.2016.58773 PMC4828508 27095958

[7] Efstathiou JA, Sampson DA, Levine Z, et al Nonsteroidal antiinflammatory drugs differentially suppress endometriosis in a murine model. Fertil Steril. 2005;83(1):171‐181. 15652904 10.1016/j.fertnstert.2004.06.058

[8] Angioni S, Cofelice V, Pontis A, Tinelli R, Socolov R. New trends of progestins treatment of endometriosis. Gynecol Endocrinol. 2014;30(11):769‐773. 25144122 10.3109/09513590.2014.950646

[9] Practice Committee of the American Society for Reproductive M . Treatment of pelvic pain associated with endometriosis: a committee opinion. Fertil Steril. 2014;101(4):927‐935. 24630080 10.1016/j.fertnstert.2014.02.012

[10] Andres MDP, Lopes LA, Baracat EC, Podgaec S. Dienogest in the treatment of endometriosis: systematic review. Arch Gynecol Obstet. 2015;292(3):523‐529. 25749349 10.1007/s00404-015-3681-6

[11] Zito G, Luppi S, Giolo E , et al Medical treatments for endometriosis-associated pelvic pain. Biomed Res Int. 2014;2014:191967. 25165691 10.1155/2014/191967 PMC4140197

[12] Granese R, Perino A, Calagna G, et al Gonadotrophin-releasing hormone analogue or dienogest plus estradiol valerate to prevent pain recurrence after laparoscopic surgery for endometriosis: a multi-center randomized trial. Acta Obstet Gynecol Scand. 2015;94(6):637‐645. 25761587 10.1111/aogs.12633

[13] Strowitzki T, Faustmann T, Gerlinger C, Schumacher U, Ahlers C, Seitz C. Safety and tolerability of dienogest in endometriosis: pooled analysis from the European clinical study program. Int J Womens Health. 2015;7:393‐401. 25926759 10.2147/IJWH.S77202 PMC4403681

[14] Brown J, Farquhar C. An overview of treatments for endometriosis. JAMA. 2015;313(3):296‐297. 25603001 10.1001/jama.2014.17119

[15] Li R-R, Xi Q, Tao L, Sheng W, Zhao C-C, Wu Y-J. A systematic review and Bayesian analysis of the adverse effects of dienogest. BMC Pharmacol Toxicol. 2024;25(1):43. 39090694 10.1186/s40360-024-00767-1 PMC11293008

[16] Veth VB, van de Kar MM, Duffy JM, van Wely M, Mijatovic V, Maas JW. Gonadotropin-releasing hormone analogues for endometriosis. Cochrane Database Syst Rev. 2023;2023(6):CD014788. 10.1002/14651858.CD014788.pub2 PMC10283345 37341141

[17] Bulun SE . Endometriosis. N Engl J Med. 2009;360(3):268‐279. 19144942 10.1056/NEJMra0804690

[18] Bulun SE, Monsivais D, Kakinuma T , et al Molecular biology of endometriosis: from aromatase to genomic abnormalities. Semin Reprod Med. 2015;33(3):220‐224. 26036904 10.1055/s-0035-1554053

[19] Klemmt PAB, Starzinski-Powitz A. Molecular and cellular pathogenesis of endometriosis. Curr Womens Health Rev. 2018;14(2):106‐116. 29861704 10.2174/1573404813666170306163448 PMC5925869

[20] Ferrero S, Barra F, Leone Roberti Maggiore U. Current and emerging therapeutics for the management of endometriosis. Drugs. 2018;78(10):995‐1012. 29946962 10.1007/s40265-018-0928-0

[21] Wongrakpanich S, Wongrakpanich A, Melhado K, Rangaswami J. A comprehensive review of non-steroidal anti-inflammatory drug use in the elderly. Aging Dis. 2018;9(1):143‐150. 29392089 10.14336/AD.2017.0306 PMC5772852

[22] Di Paola R, Fusco R, Gugliandolo E , et al Co-micronized palmitoylethanolamide/polydatin treatment causes endometriotic lesion regression in a rodent model of surgically induced endometriosis. Front Pharmacol. 2016;7:382. 27790149 10.3389/fphar.2016.00382 PMC5063853

[23] Young VJ, Ahmad SF, Duncan WC, Horne AW. The role of TGF-beta in the pathophysiology of peritoneal endometriosis. Hum Reprod Update. 2017;23(5):548‐559. 28903471 10.1093/humupd/dmx016

[24] Pizzo A, Salmeri FM, Ardita FV, Sofo V, Tripepi M, Marsico S. Behaviour of cytokine levels in serum and peritoneal fluid of women with endometriosis. Gynecol Obstet Invest. 2002;54(2):82‐87. 12566749 10.1159/000067717

[25] Lee HJ, Kim H, Ku S-Y, Kim SH, Kim JG. Transforming growth factor-beta1 gene polymorphisms in Korean women with endometriosis. Am J Reprod Immunol. 2011;66(5):428‐434. 21623988 10.1111/j.1600-0897.2011.01009.x

[26] Young VJ, Brown JK, Saunders PT, Duncan WC, Horne AW. The peritoneum is both a source and target of TGF-beta in women with endometriosis. PLoS One. 2014;9(9):e106773. 25207642 10.1371/journal.pone.0106773 PMC4160207

[27] Young VJ, Brown JK, Maybin J, Saunders PT, Duncan WC, Horne AW. Transforming growth factor-beta induced warburg-like metabolic reprogramming may underpin the development of peritoneal endometriosis. J Clin Endocrinol Metab. 2014;99(9):3450‐3459. 24796928 10.1210/jc.2014-1026 PMC4207934

[28] Hull ML, Johan MZ, Hodge WL, Robertson SA, Ingman WV. Host-derived TGFB1 deficiency suppresses lesion development in a mouse model of endometriosis. Am J Pathol. 2012;180(3):880‐887. 22210480 10.1016/j.ajpath.2011.11.013

[29] Arablou T, Aryaeian N, Khodaverdi S, et al The effects of resveratrol on the expression of VEGF, TGF-β, and MMP-9 in endometrial stromal cells of women with endometriosis. Sci Rep. 2021;11(1):6054. 33723310 10.1038/s41598-021-85512-y PMC7961000

[30] Keleş CD, Vural B, Filiz S , et al The effects of etanercept and cabergoline on endometriotic implants, uterus and ovaries in rat endometriosis model. J Reprod Immunol. 2021;146:103340. 34139652 10.1016/j.jri.2021.103340

[31] Mohankumar K, Li X, Sung N, Cho YJ, Han SJ, Safe S. Bis-indole-derived nuclear receptor 4A1 (NR4A1, Nur77) ligands as inhibitors of endometriosis. Endocrinology. 2020;161(4):bqaa027. 32099996 10.1210/endocr/bqaa027 PMC7105386

[32] Zhang L, Mohankumar K, Martin G , et al Flavonoids quercetin and kaempferol are NR4A1 antagonists and suppress endometriosis in female mice. Endocrinology. 2023;164(10):bqad133. 37652054 10.1210/endocr/bqad133 PMC10502789

[33] de Oliveira RZ, de Oliveira Buono F, Cressoni ACL, et al Overexpression of miR-200b-3p in menstrual blood-derived mesenchymal stem cells from endometriosis women. Reprod Sci. 2022;29(3):734‐742. 35075610 10.1007/s43032-022-00860-y

[34] Wu R, Li J, Li J , et al Construction of competing endogenous RNA networks incorporating transcription factors to reveal differences in granulosa cells from patients with endometriosis. Genet Test Mol Biomarkers. 2021;25(7):453‐462. 34280006 10.1089/gtmb.2020.0152

[35] Safe S, Karki K. The paradoxical roles of orphan nuclear receptor 4A (NR4A) in cancer. Mol Cancer Res. 2021;19(2):180‐191. 33106376 10.1158/1541-7786.MCR-20-0707 PMC7864866

[36] Mohankumar K, Wright G, Kumaravel S , et al Bis-indole-derived NR4A1 antagonists inhibit colon tumor and splenic growth and T-cell exhaustion. Cancer Immunol Immunother. 2023;72(12):3985‐3999. 37847301 10.1007/s00262-023-03530-3 PMC10700478

[37] Upadhyay S, Lee M, Zhang L , et al Dual nuclear receptor 4A1 (NR4A1/NR4A2) ligands inhibit glioblastoma growth and target TWIST1. Mol Pharmacol. 2025;107(2):100009. 40023516 10.1016/j.molpha.2024.100009 PMC11881746

[38] Upadhyay S, Hailemariam AE, Mariyam F, et al Bis-indole derivatives as dual nuclear receptor 4A1 (NR4A1) and NR4A2 ligands. Biomolecules. 2024;14(3):284. 38540704 10.3390/biom14030284 PMC10967861

[39] Karki K, Mohankumar K, Schoeller A, Martin G, Shrestha R, Safe S. NR4A1 ligands as potent inhibitors of breast cancer cell and tumor growth. Cancers (Basel). 2021;13(11):2682. 34072371 10.3390/cancers13112682 PMC8198788

[40] Faul F, Erdfelder E, Lang A-G, Buchner A. G*Power 3: a flexible statistical power analysis program for the social, behavioral, and biomedical sciences. Behav Res Methods. 2007;39(2):175‐191. 17695343 10.3758/bf03193146

[41] Bono Y, Kyo S, Takakura M, et al Creation of immortalised epithelial cells from ovarian endometrioma. Br J Cancer. 2012;106(6):1205‐1213. 22353808 10.1038/bjc.2012.26 PMC3304406

[42] Bankhead P, Loughrey MB, Fernández JA, et al Qupath: open source software for digital pathology image analysis. Sci Rep. 2017;7(1):16878. 29203879 10.1038/s41598-017-17204-5 PMC5715110

[43] Zhan H, Peng B, Ma J, et al Epidermal growth factor promotes stromal cells migration and invasion via up-regulation of hyaluronate synthase 2 and hyaluronan in endometriosis. Fertil Steril. 2020;114(4):888‐898. 32762950 10.1016/j.fertnstert.2020.05.005

[44] Wang Y-M, Wu M-J, Lin Y-H, Chen J. Association of epidermal growth factor receptor (EGFR) gene polymorphisms with endometriosis. Medicine (Baltimore). 2019;98(17):e15137. 31027056 10.1097/MD.0000000000015137 PMC6831181

[45] Geng R, Huang X, Li L , et al Gene expression analysis in endometriosis: immunopathology insights, transcription factors and therapeutic targets. Front Immunol. 2022;13:1037504. 36532015 10.3389/fimmu.2022.1037504 PMC9748153

[46] Dey P, Barros RP, Warner M, Ström A, Gustafsson J-Å. Insight into the mechanisms of action of estrogen receptor β in the breast, prostate, colon, and CNS. J Mol Endocrinol. 2013;51(3):T61‐T74. 24031087 10.1530/JME-13-0150

[47] Han SJ, Jung SY, Wu S-P, et al Estrogen receptor β modulates apoptosis complexes and the inflammasome to drive the pathogenesis of endometriosis. Cell. 2015;163(4):960‐974. 26544941 10.1016/j.cell.2015.10.034 PMC4640214

[48] Kazmi I, Alharbi KS, Al-Abbasi FA, et al Role of epithelial-to-mesenchymal transition markers in different stages of endometriosis: expression of the snail, slug, ZEB1, and twist genes. Crit Rev Eukaryot Gene Expr. 2021;31(2):89‐95. 10.1615/CritRevEukaryotGeneExpr.2021037996 34347983

[49] Li J, Ma J, Fei X, Zhang T, Zhou J, Lin J. Roles of cell migration and invasion mediated by twist in endometriosis. J Obstet Gynaecol Res. 2019;45(8):1488‐1496. 31250947 10.1111/jog.14001

[50] Proestling K, Birner P, Balendran S, et al Enhanced expression of the stemness-related factors OCT4, SOX15 and TWIST1 in ectopic endometrium of endometriosis patients. Reprod Biol Endocrinol. 2016;14(1):81. 27881125 10.1186/s12958-016-0215-4 PMC5122168

[51] Zeisberg M, Neilson EG. Biomarkers for epithelial-mesenchymal transitions. J Clin Invest. 2009;119(6):1429‐1437. 19487819 10.1172/JCI36183 PMC2689132

[52] Gabriel M, Fey V, Heinosalo T, et al A relational database to identify differentially expressed genes in the endometrium and endometriosis lesions. Sci Data. 2020;7(1):284. 32859947 10.1038/s41597-020-00623-x PMC7455745

[53] Mix KS, McMahon K, McMorrow JP, et al Orphan nuclear receptor NR4A2 induces synoviocyte proliferation, invasion, and matrix metalloproteinase 13 transcription. Arthritis Rheum. 2012;64(7):2126‐2136. 22275273 10.1002/art.34399

[54] Chen P, Li J, Huo Y, et al Adenovirus-mediated expression of orphan nuclear receptor NR4A2 targeting hepatic stellate cell attenuates liver fibrosis in rats. Sci Rep. 2016;6(1):33593. 27646469 10.1038/srep33593 PMC5028713

[55] Mahajan S, Saini A, Chandra V , et al Nuclear receptor Nr4a2 promotes alternative polarization of macrophages and confers protection in sepsis. J Biol Chem. 2015;290(30):18304‐18314. 25953901 10.1074/jbc.M115.638064 PMC4513091

[56] Lee SL, Wesselschmidt RL, Linette GP, Kanagawa O, Russell JH, Milbrandt J. Unimpaired thymic and peripheral T cell death in mice lacking the nuclear receptor NGFI-B (Nur77). Science. 1995;269(5223):532‐535. 7624775 10.1126/science.7624775

[57] Zetterström RH, Solomin L, Jansson L, Hoffer BJ, Olson L, Perlmann T. Dopamine neuron agenesis in nurr1-deficient mice. Science. 1997;276(5310):248‐250. 9092472 10.1126/science.276.5310.248

[58] Català-Solsona J, Miñano-Molina AJ, Rodríguez-Álvarez J. Nr4a2 transcription factor in hippocampal synaptic plasticity, memory and cognitive dysfunction: a perspective review. Front Mol Neurosci. 2021;14:786226. 34880728 10.3389/fnmol.2021.786226 PMC8645690

[59] Karki K, Li X, Jin UH, et al Nuclear receptor 4A2 (NR4A2) is a druggable target for glioblastomas. J Neurooncol. 2020;146(1):25‐39. 31754919 10.1007/s11060-019-03349-y PMC7054911

[60] Liu H, Liu P, Shi X, Yin D, Zhao J. NR4A2 protects cardiomyocytes against myocardial infarction injury by promoting autophagy. Cell Death Discov. 2018;4(1):27. 10.1038/s41420-017-0011-8 PMC5841341 29531824

[61] Gogia P, Ashraf H, Bhasin S, Xu Y. Antibody-drug conjugates: a review of approved drugs and their clinical level of evidence. Cancers (Basel). 2023;15(15):3886. 37568702 10.3390/cancers15153886 PMC10417123

[62] Burslem GM, Crews CM. Proteolysis-targeting chimeras as therapeutics and tools for biological discovery. Cell. 2020;181(1):102‐114. 31955850 10.1016/j.cell.2019.11.031 PMC7319047

